# Treatment of Cicatrices Pressure and Friction

**Published:** 1895-12

**Authors:** David Wark


### SELECTIONS.

### Treatment of Cicatrices Pressure and Friction.

When scars are situated on exposed parts of the body they are very unsightly, and when of large size are often productive of much deformity because of the strong tendency of cicatricial tissue to contract powerfully for many months after the healing process has been completed.

By means of friction and pressure persistently conducted, as here directed, the red shining surfaces of scars may be made to resemble the texture of the surrounding skin. When portions of the scar projeet above the surface these may be smoothed down, and in extensive cicatrices when tendinous bands connect the scar with underlying muscles or even with the periosteum, they can be absorbed and the natural mobility of the parts restored; moreover, the contractile tendency of extensive cicatrices may be successfully resisted and when deformity has already occurred because of such construction, it can always, at least measurably often, be quite removed by the same apparent simple means.

The treatment should be conducted as follows: If the scar be a small one, place the ends of two or three fingers on it, the hand being at an angle of thirty degrees with the surface; press firmly and vibrate the surface on the tissues beneath. The surface of the scar itself must not be subject to any friction, all the motion must be between the integument and deeper tissues.

The location of the vibratile motions and pressures should be changed every ten or fifteen seconds until the whole scar has been treated, if it be of moderate size; but if the cicatrix be large the margins only should be subjected to treatment at first; advances toward the centre must be deferred until the nutrition of the margins has been decidedly improved.

Only a little treatment should be applied to any one spot at the same time, but the pressures and vibrations should be repeated ten to fifteen times every day provided that pain and tenderness are not produced; when these are developed treatment must be suspended until they subside.

In the course of two or three weeks of such treatment the surfaces of such scars of moderate size become more mobile and will begin to form wrinkles like true skin when pressed from side to side.

The tenderness usually present will gradually subside and the natural tactile sensibility will be at least measurably restored.

All the changes are due to improved nutrition consequent on better blood circulation aided by the development of entirely new sets of blood-vessels in the parts. But no matter how successful this treatment may be in restoring the integument, hair will never grow in cicatricial tissue. — David Wark. M.D., in *National Board of Health Magazine.*

